# Acute effect of an e-cigarette with and without nicotine on lung function

**DOI:** 10.1186/1617-9625-12-S1-A34

**Published:** 2014-06-06

**Authors:** Anastasios Palamidas, Sophia Antiopi Gennimata, Georgios Kaltsakas, Stamatoula Tsikrika, Sophia Vakali, Christina Gratziou, Nikolaos Koulouris

**Affiliations:** 1 Research unit for Tobacco Control, 1st Respiratory Department, University of Athens, Medical School, Sotiria Hospital, Athens, 11527, Greece

## Background

E-cig is an electrical device that vaporizes propylene or polyethylene glycol-based liquid solution into an aerosol mist containing different concentration of nicotine. Our preliminary study showed an increase in Raw, a concomitant decrease in sGaw and an increase in slope of phase III in a limited number of subjects immediately after smoking a single e–cig containing nicotine.

## Materials and methods

We extended our protocol in a larger group of never smokers and in smokers. We implemented the same protocol with a nicotine free e-cig in a group of never smokers. We studied 60 subjects before and after smoking an e-cig containing 11mg nicotine (Group A). Group A: 9 never smokers and 51 smokers (24 had no overt airways disease, 11 asthma, 16 COPD). Another group of 10 never smokers used a nicotine free e-cig (Group B). Lung function assessed pre and post e-cig use including lung volumes, airway resistance (Raw), specific airway conductance (sGaw) and the slope of phase III. The same brand of e-cig used in both groups, with 11 and 0mg of nicotine.

## Results

Group A: a significant increase in Raw was shown in smokers and in never smokers (0.284±0.13-0.308±0.14; p= 0.033, 0.246±0.07-0.292±0.05; p=0.006) with significant decrease in sGaw (1.197±0.50-1.060±0.42; p= 0.009, 1.313±0.22-1.109±0.18; p= 0.043). Increased slope in phase III was shown only in asthmatic patients (p=0.008). Group B: increase in Raw (0.247±0.03-0.333±0.08; p=0.005) and a decrease in sGaw (1.213±0.29-0.944±0.18; p=0.009) noted.

## Conclusions

The present study supports our preliminary results showing increased Raw and a concomitant decrease in sGaw. These changes might be due to the vaporizing liquid but not to the inhaled nicotine per se.

